# Role of chromatin remodeling and immunopharmacology in cancer therapy

**DOI:** 10.1186/s10020-026-01557-7

**Published:** 2026-07-16

**Authors:** Syeda Kiran Riaz, Mehwish Iqbal, Farwa Tahir, Tariq Iqbal, Faraz Ahmad, Muhammad Saboor, Asif Naseer, Shafiul Haque, Vaibhav Srivastava

**Affiliations:** 1 Department of Molecular Biology, Shaheed Zulfiqar Ali Bhutto Medical University, Islamabad, Pakistan; 2 Office of Research, Innovation and Commercialization, Rawalpindi Medical University, Rawalpindi, Pakistan; 3 Department of Integrative Biology, School of Bio Sciences and Technology, Vellore Institute of Technology, Vellore, India; 4 Department of Medical Laboratory Sciences, College of Health Sciences, University of Sharjah, Sharjah, United Arab Emirates; 5 Research Institute for Medical and Health Sciences, University of Sharjah, Sharjah, United Arab Emirates; 6 Department of Medicine, Combined Military Hospital, Lahore, Pakistan; 7 Department of Nursing, College of Nursing and Health Sciences & Health Research Center, Jazan University, 82911 Jazan, Saudi Arabia; 8 School of Medicine, Universidad Espiritu Santo, Samborondon, 091952 Ecuador; 9 Department of Chemistry, School of Engineering Sciences in Chemistry, Biotechnology and Health, KTH Royal Institute of Technology, AlbaNova University Center, Stockholm, Sweden

**Keywords:** Chromatin Remodelers, DNA damage, Cancer, Repair, Immune system

## Abstract

Epigenetic modifications play a crucial role in cancer, influencing cellular physiology, extracellular matrix (ECM) remodeling, and immune responses. With growing emphasis on chromatin remodeling and the epigenetic regulation of immunity, these regulatory pathways and related players have become focal points of oncological investigations. Epigenetic manipulation of immune system components has emerged as a promising strategy in cancer treatment. Several FDA-approved “epi-drugs” target the epigenome to modulate immune responses and offer new opportunities across different cancers. DNA repair pathways contribute significantly to the avoidance of cancer initiation and also contribute to cellular resistance in cancer treatment. Chromatin remodelers such as INO80, Fun30, and RAD54 play essential roles in DNA damage response (DDR) through homologous recombination and non-homologous end-joining pathways, and their dysregulation leads to genome instability and tumor progression. Chromatin remodeling complexes, including SWI/SNF and CHD families, are frequently altered across various cancer types, further highlighting their role in cancer pathophysiology. Targeting these chromatin modifiers and associated DNA repair mechanisms may provide novel therapeutic options. In addition, combining epigenetic modulators with immunotherapies has shown promise in enhancing responses to immune checkpoint blockade. Epigenetic drugs such as histone deacetylase (HDAC) inhibitors and DNA methylation inhibitors are being explored for their synergistic effects with immunotherapy. While the interplay between chromatin remodeling, DNA repair, and immune responses provides a strong framework for developing targeted oncological strategies, further research is required to better understand these interactions and optimize their clinical application.

## Introduction

Cancer is a disease associated with dysregulations of the cell cycle, resulting in replicative immortality and extensive uncontrolled cellular proliferation (Hanahan and Weinberg 2011). Reprogramming of cellular properties including survival, growth, differentiation, proliferation, viability, and invasiveness are hallmarks of cancer cells. Multiple somatic alterations disrupt these core regulatory mechanisms and contribute to cancer pathophysiology. In some cases, somatic alterations directly alter gene expression resulting in enhanced tumor growth. A well-known example is the t(8;14)(q24; q32) translocation, which results in overexpression of Myc protein (Erikson et al. 1983). Certain mutations induce widespread changes in gene expression, affecting multiple components of regulatory gene circuits that control various aspects of cellular physiology. Activating mutations in the Ras gene (RasG12V) that stimulate pro-tumorigenic signaling pathways exemplify this mechanism (Ayllón et al., 2000). Abnormal gene expression can sustain the malignant phenotype by altering oncogenic, immune and stress response pathways, a phenomenon referred to as “oncogene addiction” (Weinstein 2002).

Research in oncological epigenetics indicates extensive reprogramming of multiple components of epigenetic machinery across various cancer types. Epigenetic alterations play critical roles in breast cancer initiation and metastasis. During cancer progression, the extracellular matrix (ECM) undergoes marked structural changes, accompanied by induction of epithelial-to-mesenchymal transition (EMT). Altered expression of ECM molecules can lead to matrix remodeling, which exerts regulatory effects on tumor cell pathophysiology, influencing proliferation, migration, invasion, and EMT. These processes are closely associated with epigenetic regulation (Zhou and Yu 2024). Studies have shown that epigenetic modifications and regulators, such as DNA methylation, nucleosome repositioning, and non-coding RNAs, play critical roles in triple-negative breast cancer (TNBC) prognosis. Indeed, multiple aspects of ECM and EMT dysregulation in tumor progression are associated with epigenetic alterations, indicating the pleiotropic nature of these mechanisms in cancer (Weinstein 2002).

Given this backdrop, there is a need for focused elucidation of the complex interactions between tumor cells, immune modulators, and host response systems. Unsurprisingly, the potential to exploit immune regulators for the effective treatment of various carcinomas has gained considerable attention in recent years. The development, evaluation, and refinement of novel immunotherapeutic approaches targeting tumor cells are actively ongoing. This review first provides insights into the epigenetic regulation of DNA damage and immune responses in cancer, with particular emphasis on chromatin remodelers. It then discusses key immunotherapeutic strategies currently employed in cancer management.

## DNA repair process

### Overview

DNA damage arises from multiple intrinsic and extrinsic factors and, if left unrepaired, it can cause either cell death or various types of mutation leading to tumorigenesis. Among various forms of DNA damage, DNA double-strand breaks (DSBs) are considered to be the most serious, as even one unrepaired DSB can result in cell death. Endogenous DNA repair mechanisms restore DNA integrity, although preservation of precise genetic information is not always guaranteed. Evolutionary conserved repair pathways for DSBs include homologous recombination (HR) and non-homologous end joining (NHEJ) (Hou et al. 2024; Prime et al. 2024).

Cyclin-dependent kinases (CDKs) regulate DSB end resection by activating nucleases involved in DNA end processing. This activity generates single-stranded DNA (ssDNA) and enables the recruitment of repair and checkpoint proteins to the sites of damage (Janke et al. 2010). The extent of DSB end resection (5′−3′ trimming that produces 3′ overhangs) determines the choice of DSB repair pathway. During the S and G2 phases, resection is upregulated and favors HR. In contrast, during the G1 phase, resection remains limited and promotes NHEJ. HR is generally considered a high-fidelity pathway, whereas NHEJ is more error-prone. NHEJ is mediated by Ku70/Ku80, DNA-dependent protein kinase, catalytic subunit (DNA-PKcs), ligase IV, and X-ray repair cross-complementing protein 4 (XRCC4) (Dip and Naegeli 2005). The 3′ overhangs generated by resection are rapidly bound by replication protein A (RPA), forming an ssDNA-RPA complex that promotes recruitment of DNA repair machinery. Activation of the Mec1-Ddc2 damage-sensing checkpoint kinase initiates checkpoint signaling (San Filippo et al. 2008). In HR, initial resection is mediated by the MRX complex (MRE11-RAD50-XRS2) together with the endonuclease SAE2, generating ssDNA. RPA subsequently binds ssDNA, followed by recruitment of RAD51 to form a nucleoprotein filament that mediates homology search within the sister chromatid. DNA polymerase extends the invading strand, leading to Holliday junction formation, which is later resolved and ligated to complete the repair process. Additional nucleases recruited by MRX include DNA2, exonuclease 1 (EXO1), and SGS1, facilitate extensive ssDNA generation. These enzymes are critical for robust DNA damage checkpoint activation (D’Amours and Jackson 2002). MRE11 possesses both endonuclease and 3′−5′ exonuclease activity. Although its catalytic activity is not required for repair of HO endonuclease-induced DSBs, it is essential for resolving DNA adducts and protecting against ionizing radiation (Llorente and Symington 2004). SAE2 has been proposed as a key factor in HO endonuclease-induced DSB repair, although direct physical interaction with MRX remains unclear. Both MRE11 and SAE2 participate in processing mitotic DSB ends with hairpin-like structures and contribute to initial end resection (Lengsfeld et al. 2007; Lobachev et al. 2002). SAE2 activity depends on CDK1-mediated phosphorylation at Ser267; mutation at this residue (S267A) abolishes phosphorylation and mimics SAE2 deletion (∆*sae*2) (Huertas et al. 2008). Cells lacking SAE2 or harboring MRX mutations exhibit delayed resection initiation near DSB sites, though distal resection remains largely unaffected (Mimitou and Symington 2008). Similarly, deletion of DNA2, EXO1, or SGS1 impairs extensive end resection, particularly within 100–200 bp of the break site. Initial resection depends primarily on MRX and SAE2, which clear nucleotides at DSB ends to permit further processing. Once resection is initiated, it can proceed in the absence of MRX, suggesting these proteins accelerate but are not absolutely required for the continuation of resection (Zhu et al. 2008). The second phase involves long-range resection mediated by EXO1 and the SGS1/DNA2 pathway. EXO1 is a 5′−3′ exonuclease implicated in DSB resection, mismatch repair, and UV-induced DNA damage processing (Tran et al. 2004). Its activity is regulated by phosphorylation (Morin et al. 2008). Although ∆exo1 mutants are not highly sensitive to ionizing radiation, they exhibit reduced resection rates compared with wild-type cells. Genetic interactions between EXO1, MRE11, and SAE2 indicate cooperative functions; double mutants (∆mre11/∆exo1 or ∆sae2/∆exo1) display more severe defects in DSB resection and increased sensitivity to DNA-damaging agents (Moreau et al. 2001). Further, ∆mre11/∆exo1 and ∆sae2/∆exo1 double mutants show resection and HR pathway activation, indicating the possibility for some additional pathways in end resection. Indeed, SGS1, a 3′−5′ RecQ helicase, is known to contribute to additional resection pathways. Combined inactivation of SGS1 and EXO1 abolishes DSB resection (Mimitou and Symington 2008). SGS1 forms a complex with DNA topoisomerase III (TOP3) and RMI1 (the STR complex), which enhances helicase efficiency and promotes DSB processing. Following SGS1-mediated ssDNA generation, DNA2 further processes DNA ends, with CDK1-dependent phosphorylation facilitating its recruitment to DSB sites (Chen et al. 2011).

### DSB repair in cancer pathogenesis

DNA damage response (DDR) pathways are critical for protecting cells from genomic instability and mutations. Erroneous or dysregulated DNA repair can cause chromosomal translocations, genomic instability, and accumulation of mutations. Such dysfunctions in DDR mechanisms significantly contribute to cancer initiation, progression, and metastasis (Powell and Bindra 2009). Cancer cells exhibit altered checkpoint activation, redox regulation, and DNA repair pathways. Therefore, the interplay between cancer and DDR pathways provides a rationale for targeting these pathways using specific inhibitors, particularly in tumors with hyperactive repair mechanisms during chemotherapy (Lee et al. 2025; Marcut et al. 2025).

Below, we discuss some of the components of the DDR pathways known to be associated with metastases. MutT homolog 1 (MTH1), a protein involved in maintaining dNTP pool integrity, has been associated with multiple cancers (Huber et al. 2014). MUC1-C, an oncoprotein implicated in DDR, is expressed in over 90% of TNBC cases. Targeting MUC1-C reduces poly(ADP-ribose) polymerase 1 (PARP1) levels within the DDR pathway and demonstrates synergistic effects with the PARP inhibitor olaparib in TNBC cellular models (Yamamoto et al. 2019). Mutations in HR genes are strongly linked to tumorigenesis. Germline mutations in BRCA1 and BRCA2, which are central to HR-mediated repair, are well characterized in hereditary breast and ovarian cancers (Petrucelli et al. 2010). Secondary mutations at these loci may restore open reading frames (ORFs) and re-express functional protein, leading to acquired resistance to chemotherapeutic agents such as cisplatin (Helleday 2010; Dhillon et al. 2011). RAD51, another key HR protein, is frequently overexpressed in various human cancers (Hannay et al. 2007). Elevated RAD51 levels have been associated with enhanced DNA repair capacity and resistance of small-cell lung cancer cells to cytotoxic agents such as etoposide (Hansen et al. 2003). Reduced expression of HR-associated proteins, including MRE11, RAD50, and NBS1 (the MRN complex), has also been reported in breast cancers. In fact, dysregulation of MRN components appears to be a common feature underlying multiple malignancies (Mosor et al. 2006).

Hyperactivation of the NHEJ damage repair pathway has likewise been implicated in therapeutic resistance (Begg et al. 2011). Chemoresistant murine breast cancer cells exhibit reduced γH2AX foci formation following exposure to 5 Gy γ-radiation, suggesting enhanced DSB repair capacity (Li et al. 2008). Increased expression of Ku proteins and DNA-PKcs has been observed in several cancers (Beskow et al. 2009). Elevated Ku levels, coupled with increased DNA-binding activity in radiation-resistant cancer cells, further support the presence of hyperactive DNA repair systems (Frit et al. 1999). Apart from the impact on the fate of radiotherapy outcomes, other modes of tumor treatment are also affected by NHEJ proteins. Because of the key roles of NHEJ and HR repair pathways in promoting tumorigeneses (Table 1), downregulating their activities may be targeted as strategies to enhance the sensitivity of cancer cells to radio- and chemotherapeutic processes.

**Table 1:** Proteins belonging to HR and NHEJ pathways of double-strand break repair and their involvement in different cancers

Protein	Pathway	Alteration in Cancer	Cancer Association	Clinical Implications
RAD51	HR	Overexpressed	Breast, gastric, oral, lung, esophageal carcinomas, leukemia (Sishc and Davis 2017)	Elevated RAD51 confers chemoresistance; target for synthetic lethality
BRCA1	HR	Germline/somatic LOF mutation	Hereditary breast and ovarian cancer (Petrucelli et al. 2010)	PARP inhibitor sensitivity; predictive biomarker for platinum response
BRCA2	HR	Germline/somatic LOF mutation	Hereditary breast, ovarian, pancreatic, prostate cancer (Petrucelli et al. 2010)	PARP inhibitor sensitivity; reversion mutations cause cisplatin resistance
MRE11	HR	Reduced expression/mutation	Breast cancer, melanoma, colorectal, head and neck cancers (Park et al. 2011; Ziółkowska-Suchanek et al. 2013)	MRN disruption impairs HR; associated with genomic instability
RAD50	HR	Reduced expression	Breast cancer, and head and neck cancers (Park et al. 2011; Ziółkowska-Suchanek et al. 2013)	HR deficiency; potential synthetic lethality with PARP inhibitors
NBS1	HR	Reduced expression/germline mutation	Breast cancer, acute lymphoblastic leukemia (Masi and Antoccia 2008)	Cancer predisposition and radiosensitivity
MRN complex	HR	Altered expression/mutation	Melanoma, ovarian, colorectal, head and neck cancers (Ziółkowska-Suchanek et al. 2013)	MRN disruption leads to HR deficiency and therapy resistance
KU70/XRCC6	NHEJ	Overexpressed	Radioresistant cervical cancer, leukemia (Beskow et al. 2009; Frit et al. 1999)	Overexpression correlates with radioresistance; KU inhibitors under preclinical investigation
DNA-PKcs	NHEJ	Overexpressed	Cervical cancer (Beskow et al. 2009)	DNA-PKcs inhibitors (e.g., peposertib) in clinical trials; radiotherapy sensitivity
XRCC4	NHEJ	Reduced expression/mutation	Breast, lung, colorectal cancers (Emami et al. 2015; Yu et al. 2023)	Loss impairs NHEJ; associated with genomic instability and cancer susceptibility
Ligase IV	NHEJ	Mutation (LIG4 syndrome)	T-cell lymphoma, hematological malignancies (Altmann and Gennery 2016)	Extreme radiosensitivity; ligation step is a potential therapeutic target

*Abbreviations*: *BRCA1* breast cancer gene 1, *BRCA2* breast cancer gene 2, *DNA-PKcs* DNA-dependent protein kinase catalytic subunit, *HR* homologous recombination, *KU70/XRCC6* X-ray repair cross-complementing protein 6, *LIG4* DNA ligase IV, *LOF* loss-of-function, *MRE11* meiotic recombination 11 homolog, *MRN* MRE11–RAD50–NBS1, *NBS1* Nijmegen breakage syndrome 1, *NHEJ* non-homologous end joining, *PARP* poly (ADP-ribose) polymerase, *RAD50* RAD50 double-strand break repair protein; RAD51, RAD51 recombinase, *XRCC4* X-ray repair cross-complementing protein 4

### DNA damage-induced immune response

Deficiencies in DNA repair pathways have been associated with enhanced responses to immunotherapies, leading to the exploration of potential predictive biomarkers for response to immune checkpoint blockade therapy. The interaction between the immune system and tumor development has become a focal point in cancer research, particularly with the recognition of the tumor microenvironment (TME) and its impact on immune cell infiltration and function (Ciner et al. 2025; Xu et al. 2023). Tumor cells evade immune surveillance by upregulating inhibitory pathways such as cytotoxic T-lymphocyte-associated protein 4 (CTLA-4) and programmed cell death protein 1/programmed cell death ligand 1 (PD-1/PD-L1), while downregulating pathways that stimulate immune responses, fostering immune escape from the TME (Tufail et al. 2025). Hypermutation and genomic instability can lead to the generation of neoantigens that engage antitumor immunity. In addition, cytoplasmic DNA fragments resulting from DDR deficiencies activate the cGAS-STING pathway, driving type-I interferon responses and promoting immune cell recruitment, which has emerged as a crucial mechanism in immune activation and response to immunotherapies (Shen et al., 2025). Understanding these intricate interactions between the DDR, immune activation, and therapeutic strategies is essential for the development of effective cancer therapies (Barros et al. 2022).

### Tumor-associated antigens (TAAs) in immune responses and epigenetic modulation

TAAs are indispensable for the induction of antitumor immunity. However, tumor cells can evade host immune surveillance by disrupting the antigen presentation machinery, thereby eliminating one of the key mechanisms required for effective immune recognition. Efficient activation of the immune response, and consequently successful immunotherapy, depends on intact antigen processing and presentation pathways (Waibl Polania et al. 2025). Presentation of TAAs requires proper expression of major histocompatibility complex (MHC) molecules and co-stimulatory signals to ensure effective activation of effector T-cells (Zhou et al. 2020). An effective antitumor response requires a very large percentage of antigen-specific CD8 + T-cells. Low or absent antigen presentation by cancer cells fall below the threshold level for detection by immune systems, which represent routes for immune evasion. In addition to other cellular mechanisms, the expression of TAAs in different tumor cells is under strong epigenetic regulation (Correia et al. 2021).

#### Epigenetic strategies to enhance TAA expression and immune regulation

Clinically, DNA methyltransferase (DNMT) inhibitors have been shown to enhance tumor immunogenicity and immune recognition by reactivating the expression of TAAs. Accordingly, inhibitors targeting epigenetic modification enzymes are increasingly being tested for oncological purposes (Table 2). DNMT inhibition has been reported to upregulate the expression of MHC class I and class II molecules in several cancers (Manning et al. 2008). For example, decitabine, a DNMT inhibitor, enhances MHC class I and II expression in chronic lymphocytic leukemia cell lines, thereby increasing interferon-γ production by tumor-specific cytotoxic T lymphocytes. DNA methylation serves as a key regulatory mechanism controlling the expression of cancer-testis antigens (CTAs), a subgroup of tumor-specific antigens. For instance, 5-azacytidine (5-AzaC) upregulates CTA expression and promotes pro-inflammatory functions of dendritic cells by increasing the expression of MHC class I and II molecules, as well as co-stimulatory markers including CD80, CD86, CD205, and CD40 (Bai et al. 2022; Li et al. 2024). Other studies have demonstrated that a variety of CTAs are aberrantly expressed in different tumor cell lines, including malignant glioma and melanoma, following treatment with DNA demethylating agents. These findings suggest that epigenetic silencing plays a central role in regulating CTA expression (Lu et al. 2025). Recent evidence further indicates that enhancer of zeste homolog 2 (EZH2) is overexpressed or aberrantly activated in melanoma and several other malignancies. Increased EZH2 activity contributes to epigenetic repression of antigen presentation machinery and tumor suppressor–associated genes, thereby promoting immune evasion and tumor progression (Gilardini Montani et al. 2024). These findings underscore the therapeutic potential of epigenetic drugs in reversing tumor immune evasion by restoring TAA expression and antigen presentation, thereby sensitizing tumors to immunotherapy.

**Table 2:** Epigenetic modification enzyme inhibitors used in cancer treatment (Li et al. 2024)

Name	Mechanism of Action	Clinical Applications
Azacitidine	DNMT inhibitor	AML, CMML, MDS
Decitabine	DNMT inhibitor	AML, CMML, MDS
Vorinostat	HDAC inhibitor	CTCL
Romidepsin	HDAC inhibitor	CTCL, PTCL
Belinostat	HDAC inhibitor	PTCL
Panobinostat	HDAC inhibitor	Multiple myeloma
Chidamide	HDAC3 inhibitor	PTCL
Enasidenib	IDH2 inhibitor	AML

*Abbreviations*: *AML* acute myeloid leukemia, *CMML* chronic myelomonocytic leukemia, *CTCL* cutaneous T cell lymphoma, *DNMT* DNA methyltransferase, *HDAC* histone deacetylase, *IDH* isocitrate dehydrogenase, *MDS* myelodysplastic syndromes, *PTCL* peripheral T-cell lymphoma

Cellular immunity is initiated through the recruitment of effector T-cells into the TME, accompanied by the release of pro-inflammatory cytokines. With regards to epigenetic regulation of tumor immune evasion, trimethylation of histone H3 at lysine 27 (H3K27me3), mediated by the Polycomb repressive complex 2 (PRC2) component EZH2, suppresses effector T-cell trafficking by reducing the expression of TH1-type chemokines CXCL9 and CXCL10. Interestingly, epigenetic-based pharmacological therapy restores chemokine expression, increases intratumoral effector T-cell infiltration, and improves the therapeutic efficacy of PD-L1 checkpoint blockade, supporting a role for epigenetic silencing in tumor immune evasion (Peng et al. 2015; Silasi et al. 2020).

#### Role of the TME in immunosuppression

Tumor progression is strongly shaped by immuno-epigenetic regulation of immune cell function within the TME. Immune cell plasticity, inflammation, and immunosuppression collectively drive tumor development. Effective tumor control therefore requires modulation of immune functions and cytotoxic activity of effector cells. Key cellular mediators within the TME include myeloid-derived suppressor cells (MDSCs), immature dendritic cells (DCs), tumor-associated macrophages, and regulatory T-cells (Tregs) (Lange-Consiglio et al. 2020; Giallongo et al. 2014). Within the TME, immunosuppressive components are known to impair T-cell activation, migration, and proliferation. Epigenetic modifications can alter immune cell function and shift the immune landscape toward immunosuppressive cell populations. Aberrant epigenetic regulation has been associated with increased accumulation of MDSCs and Tregs, which support tumor progression and the establishment of pre-metastatic niches. Extensive alterations in the epigenetic landscape may also drive polarization of neutrophils and macrophages toward the N2 and M2 immunosuppressive phenotypes, respectively, thereby promoting tumorigenesis in many cancers (Pan et al. 2020). These changes suppress acute inflammatory responses and promote infiltration of MDSCs and Tregs, leading to increased secretion of anti-inflammatory cytokines and chemokines (Salminen 2020). Treg lineage specification, gene expression, lineage stability, and immunosuppressive function are governed by Treg-specific epigenetic modifications, including stable DNA demethylation at loci such as *CTLA4*, *IKZF4*, and *TNFRSF18*. EZH2 has been shown to promote peripheral Treg differentiation and function and may influence responses to PD-1-targeted immunotherapy. Furthermore, epigenetic regulation of macrophage polarization states can modulate T-cell infiltration and antitumor immune responses within the TME (Nasrallah et al. 2020). Epigenetic drugs such as EZH2 inhibitors can target multiple immune cell populations; and reduce the production and accumulation of MDSCs and inhibit the differentiation and suppressive functions of Tregs (Mabe et al. 2024; Zheng et al. 2025; Stone et al. 2017). Thus, epigenetic therapies represent promising strategies to enhance T-lymphocyte infiltration into tumors and to improve the efficacy of ongoing immunotherapies.

## Chromatin remodeling processes

### Overview

**Fig. 1:**
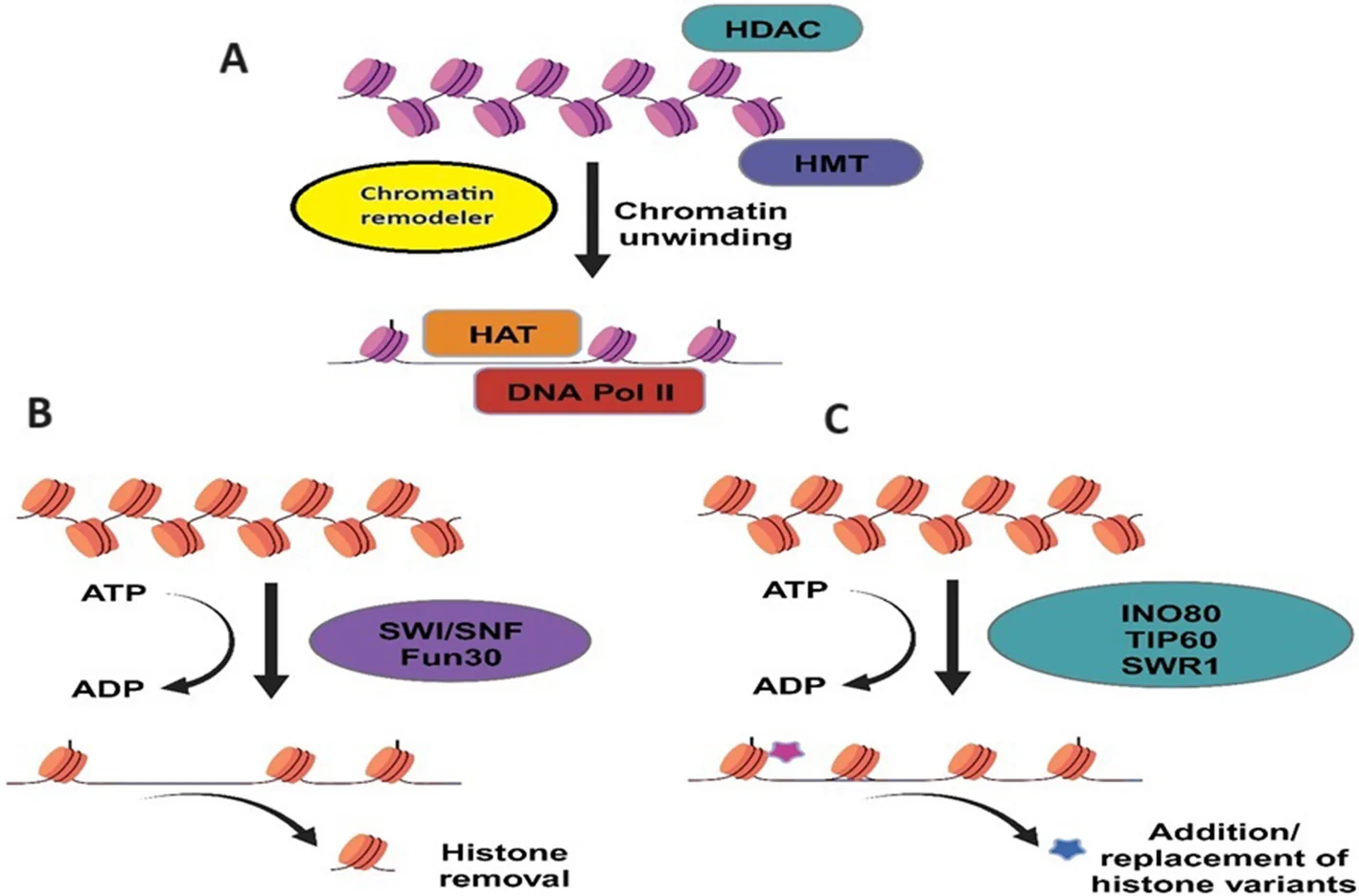
Chromatin unwinding in DNA repair & Role and mechanism of action of different chromatin remodelers. Histone methylase (HMT) and histone deacetylase (HDAC) are involved in the formation of heterochromatin. On the other hand, chromatin remodelers unwind densely packed chromatin to open configuration along with the help of histone acetyltransferases (HAT). Chromatin remodelers can be divided into six major families: Swi2/Snf2-like, Swr1-like, SMARCAD1, Rad54-like, Rad5/16-like and ERCC6/SSO1653-like (Morin et al. 2008). DNA polymerase II (DNA Pol II) plays a prominent role in the repair process but can only access DNA sequences after unwinding

Nucleosomes are the fundamental structural units of chromatin, consisting of approximately 146 base pairs of DNA wrapped around a histone octamer. Histones are multimeric proteins composed of distinct subunits. The nucleosomal core is formed by two H2A-H2B dimers and one H3-H4 tetramer, around which DNA is tightly wound (Luger et al. 1997). DNA nucleosomes are highly complex structures which regulate the interaction of DNA-binding proteins with DNA sequences. This configuration can be altered primarily in a two-step manner to allow physical interaction and binding of proteins with DNA (Eberharter et al. 2005). The first group of enzymes involved in chromatin unwinding drive post-translational modifications (e.g., methylation, acetylation, ubiquitination and phosphorylation) in histone in the core region or the N-terminal tails. These modifications can subsequently act as signals for identification of the downstream components which catalyze relaxation of the chromatin (Jenuwein and Allis 2001). This second group of enzymes are ATP-driven chromatin remodelers which use energy generated from ATP hydrolysis to physically unwind nucleosomes. The process of chromatin remodeling has been aptly described as the “access, repair, restore” concept in the repair of damaged DNA (Green and Almouzni 2002). According to this model, chromatin structure is altered at the DNA damage points, allowing access to DNA repair machinery (Fig. 1). Following repair, chromatin is restored in its original form.

### Mode of action of different chromatin remodelers at DNA damage sites

**Fig. 2:**
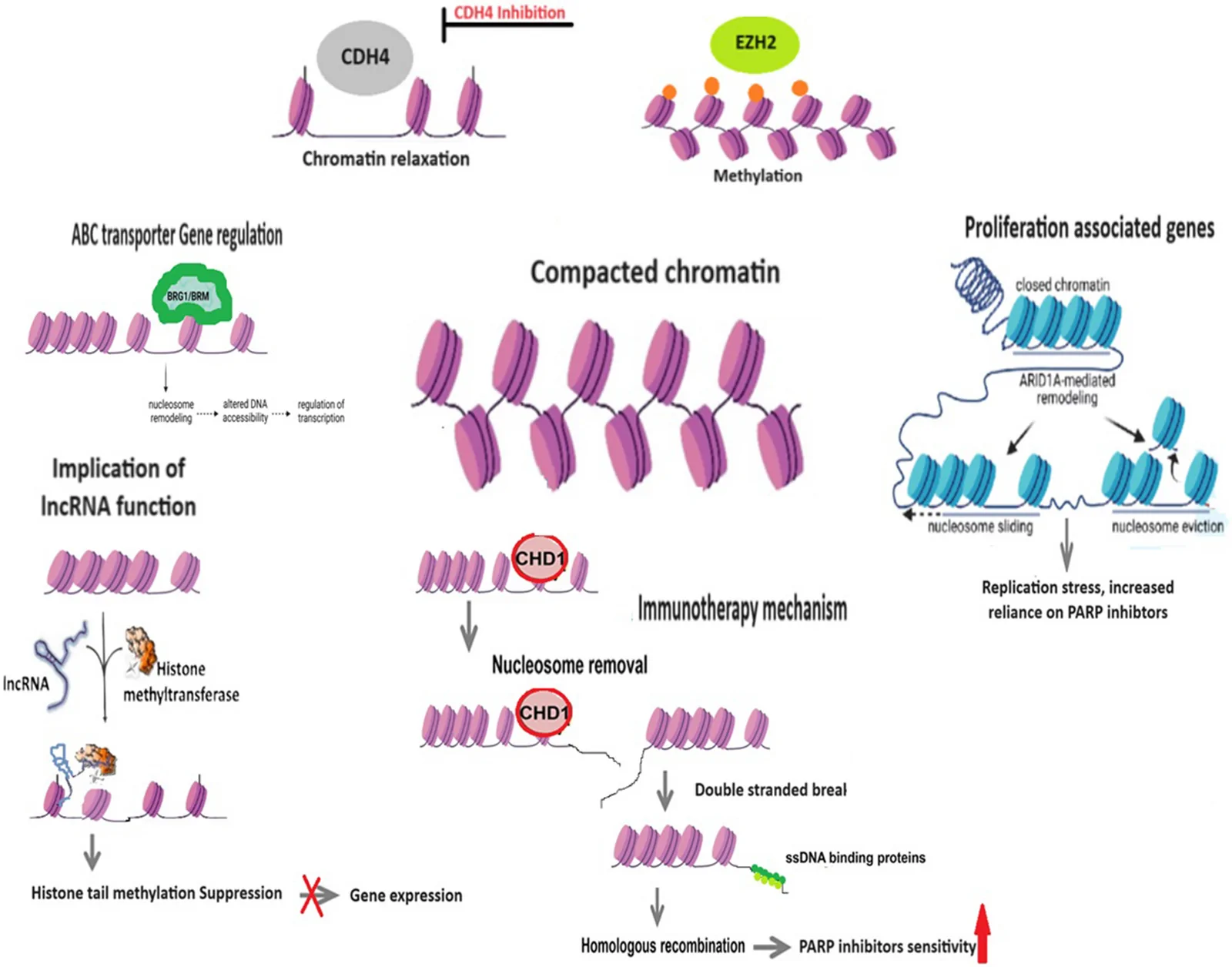
Chromatin remodeling complexes regulate gene expression, genomic stability, and therapeutic vulnerability in cancer. Chromodomain helicase DNA-binding protein 4** (**CDH4) promotes chromatin relaxation which is inhibitable pharmacologically, while EZH2 mediates repressive histone methylation. Brahma-related gene 1/Brahma (BRG1/BRM) regulates ABC transporter genes via nucleosome remodeling; lncRNA-directed histone methyltransferase recruitment suppresses gene expression through histone tail methylation. Chromodomain helicase DNA-binding protein 1 (CHD1)-mediated nucleosome removal from compacted chromatin generates double-strand breaks that are channeled into HR, sensitizing cells to PARP inhibitors. ARID1A similarly remodels closed chromatin at proliferation-associated genes via nucleosome sliding/eviction, inducing replication stress and PARP-inhibitor dependence

Previous studies have shown that downregulation or mutation of specific subunits of chromatin remodeling complexes increases cellular sensitivity to DNA-damaging agents (Chambers and Downs 2012). Multiple lines of evidence suggest that many chromatin remodelers directly participate in the DNA repair process. The roles of selected chromatin modifiers in the repair of DSB sites are summarized in Fig. 2.

In subsequent subsections, we delineate the major chromatin remodelers and their ability to alter oncological pathophysiology. The chromatin remodelers discussed including INO80, SMARCAD1/Fun30, SWI/SNF, TIP60, SWR1, and RAD54, were selected because they represent the best-characterized exemplars across the major ATP-dependent chromatin remodeling families, with direct and mechanistically established roles at DSB sites, supported by extensive genetic, biochemical, and cell biological evidence from both yeast and mammalian systems. The aim here is not to provide an exhaustive catalog of all remodelers implicated in the DDR, but rather use these well-studied exemplars to illustrate the mechanistic principles by which chromatin remodeling facilitates DSB repair via both homologous recombination (HR) and non-homologous end joining (NHEJ) pathways. The therapeutic relevance of these remodelers is addressed in Sect. "Chromatin remodelers in cancer pathophysiology". Notably, mammalian SWI/SNF complexes facilitate DSB repair by promoting γH2AX induction (Park et al. 2006), and the ISWI-family complex ACF1 is specifically required for NHEJ of DSBs in human cells (Lan et al. 2010). A cooperative activation loop among SWI/SNF, γH2AX, and H3 acetylation operates at DSB sites (Lee et al. 2010), and histone acetylation by CBP/p300 at break sites further facilitates SWI/SNF remodeling and NHEJ factor recruitment (Ogiwara et al. 2011). SWI/SNF subunits ARID1A and ARID1B show interdependent protein stability and are required for cellular resistance to DNA damage (Watanabe et al. 2014). More recently, BRD3 was shown to initiate DSB-induced chromatin remodeling with TIP60 to promote R-loop-mediated HR (Qian et al. 2025).

#### INO80

INO80 remodeler is a member of the INO80 subfamily within the broader INO80/SWR1 superfamily of remodelers, with a split ATPase domain with a large central insertion (Clapier and Cairns 2009). INO80 is recruited at DSBs in both yeast and humans and influences the functioning of both the HR and the NHEJ repair pathways (Tsukuda et al. 2005). When a DSB is generated, cells arrest themselves in the G2 phase, so that the repair machinery can act on the damaged DNA. Cells exit checkpoint arrest only once the repair process is completed. In yeast, INO80 complex is made up of 15 subunits, and unlike the wild-type cells, *ino80*∆ cells remain arrested showing failure to adapt on checkpoint activation (Papamichos-Chronakis et al. 2006; Lee et al. 1998). Phosphorylation of H2A histone is thought to play a critical role in the recruitment of INO80 at DSB sites, and mutations in H2A reduce the binding of INO80 at damage site (Attikum et al. 2004). INO80 prevents the exchange between γH2AX-H2AZ at the damage site, by depositing H2A that can be phosphorylated by Mec1/Tel1 kinase (Downs et al. 2000). Thus, INO80 downregulates the replacement of γH2AX for H2AZ mediated by chromatin remodeler SWR1. The model that states that γH2AX is necessary for checkpoint adaptations is seemingly at odds with the finding that failure to dephosphorylate γH2AX delays checkpoint recovery in cells with repairable DSBs (Keogh et al. 2006). Non-essential INO80 chromatin remodeling complex subunit (NHP10) is involved in this interaction and actin-related protein (ARP4) shows physical interaction with γH2AX (Downs et al. 2004). Loss of any catalytic subunit of INO80, especially the ATPase subunit or the ATPase stimulating ARP8/5 subunit, increases the sensitivity toward DNA distorting agents. Interestingly, 3’to 5’ DNA end resection in HR may be deficient in arp∆ mutants (Attikum et al. 2004). On the other hand, resection processing of DNA has been shown to be similar in wild-type and *arp*∆ mutants, however, in the mutants, loading of RAD52 and RAD51 is delayed (Tsukuda et al. 2005). From a translational perspective, INO80 subunits RUVBL1 and RUVBL2 are overexpressed in hepatocellular carcinoma and colorectal cancer, and INO80 represents an emerging candidate for targeted therapeutic strategies (see Sect. "Chromatin remodelers in cancer pathophysiology").

#### SMARCAD1/Fun30

Fun30 is a relatively less well-characterized chromatin remodeler belonging to the Etl1/Snf2-like family. Its best-described role involves the maintenance of heterochromatin at centromeres, telomeres, and the mating-type (MAT) locus in yeast (Durand-Dubief et al. 2012). The ATPase activity of Fun30 is stimulated upon binding to DNA or chromatin, and it contributes to suppression of transcriptional noise at centromeric regions. Genome-wide binding and nucleosome positioning analyses have shown that Fun30 is enriched at centromeres. Moreover, deletion of *FUN30* alters the occupancy of the histone variant H2A.Z (Htz1) at these sites. Fun30 activity may be regulated by Cdk1-mediated phosphorylation at serine 28. Cells expressing non-phosphorylatable mutants of Fun30 exhibit defective checkpoint adaptation. In addition, phosphorylation of histone H2A at serine 129 influences Fun30 recruitment to sites of DNA damage. In vitro studies suggest that Fun30 preferentially binds the non-phosphorylated form of H2A (Chen et al. 2012), indicating that phosphorylated H2A may indirectly modulate Fun30 recruitment. Recruitment of Fun30 to DSB sites is markedly reduced in *∆mec1/∆tel1/∆sml1* mutants, which exhibit impaired checkpoint activation, as well as in *∆sgs1/∆exo1* mutants which are deficient in end resection (Costelloe et al. 2012). Thus, it can be postulated that phosphorylated H2A might play an indirect role in Fun30 recruitment.

SMARCAD1, the human ortholog of Fun30, is recruited to DSB sites and co-localizes with γH2AX. Downregulation of SMARCAD1 impairs DSB end resection and processing, and reduces the formation of RPA-ssDNA foci. Cells lacking SMARCAD1 display defects in HR and increased sensitivity to DNA-damaging agents such as camptothecin and the PARP inhibitor ABT-888 (Costelloe et al. 2012). Although no clinical-stage inhibitors of SMARCAD1/Fun30 currently exist, its critical role in promoting HR-mediated resection positions it as a potential synthetic lethality target in HR-deficient tumors, with SMARCAD1 depletion conferring sensitivity to PARP inhibitors.

#### SWI/SNF

The SWI/SNF complex in yeast is composed of 11 subunits, including the catalytic ATPase subunit Swi2/Snf2. This complex is conserved across eukaryotes and belongs to the family of ATP-dependent chromatin remodeling proteins. SWI/SNF is recruited to the sites of DNA damage, where it plays an important role in DNA repair. Swi2/Snf2 subunits are rapidly recruited to damaged sites following induction of double-strand breaks (DSBs). In mammalian cells SWI/SNF influences both NHEJ and HR by facilitating nucleosome repositioning and eviction (Sinha et al. 2009). While mutants lacking the Snf2 subunit do not exhibit increased sensitivity to UV or ionizing radiation (Shen et al. 2000), other studies have reported that yeast strains with *SNF2* and *SNF5* deletions are sensitive to bleomycin and hydroxyurea, a replication-stalling compound (Chai et al. 2005). SWI/SNF associates with both the recipient and donor loci, MAT*a* and HML*a*, respectively. Deletion of the SWI/SNF remodeler disrupts synapsis between HMLa DNA and the invading MATa ssDNA. The strand annealing protein Rad52 and the strand invasion protein Rad51 are recruited to the recipient MAT locus with normal kinetics in *swi/snf* mutants; however, Rad52 and Rad51 fail to associate with the HMLa donor locus (Sinha et al. 2009). In vitro studies suggest that chromatin remodeling mediated by SWI/SNF alters histone-DNA interactions, enabling nucleosome sliding and remodeling, and histone dimer exchange (Johnson et al. 2005). Indeed, SWI/SNF-mediated chromatin remodeling has been shown to be required for Asf1-associated nucleosome displacement at the activated promoter of the *PHO5* gene (Adkins et al. 2004). However, the precise role of the SWI/SNF complex in strand exchange during HR has not been fully elucidated. Both HMRa and HMLa, which are targets of SWI/SNF remodeling activity, are organized into heterochromatic structures that form condensed and folded chromatin, making them refractory to many DNA–protein interactions (Weiss and Simpson 1998). During mitosis, SWI/SNF is required for transcription of inducible genes (Krebs et al. 2000). Therefore, SWI/SNF may facilitate HR repair by disrupting chromatin architecture at HM donor loci, thereby promoting DNA-DNA and protein-DNA interactions required for synaptic filament formation (Varga-Weisz and Becker 2006).

In humans, the SWI/SNF complex contains two catalytic subunits, Brahma-related gene 1 (BRG1) or Brahma (BRM), along with several BRG1/BRM-associated factors (BAFs). This complex has been implicated in γH2AX spreading and histone acetylation at DSB sites. The bromodomain of the BRG1 subunit recognizes acetylated histone H3 at the sites of DNA damage. SWI/SNF is thought to enhance H2AX phosphorylation, leading to increased histone acetylation (Lee et al. 2010). Additionally, SWI/SNF recruitment to DSB sites may be facilitated by the early DDR protein BRIT1/MCPH1, which promotes ATR/ATM-dependent phosphorylation of the SWI/SNF subunit BAF170. Further, depletion of BRIT1/MCPH1 has been associated with impaired chromatin relaxation during both HR and NHEJ repair pathways. These findings establish SWI/SNF as a key regulator of both HR and NHEJ, with direct therapeutic relevances; loss-of-function mutations in BRG1 (SMARCA4) and ARID1A are among the most frequent cancer alterations, and SWI/SNF subunit status is an emerging predictive biomarker for PARP inhibitor and EZH2 inhibitor sensitivity (Peng et al. 2009).

#### TIP60

TIP60 is a multi-subunit, evolutionarily conserved chromatin remodeling complex with ATPase activity (Li et al. 2024). It functions as a tumor suppressor and participates in transcriptional activation as well as in DSB repair (Santopietro et al. 2025). Initially isolated from human cells, the TIP60 complex has been reported to consist of approximately 16 subunits. It exhibits Esa1-like histone acetyltransferase (HAT) activity, ATPase activity, DNA-binding capacity, and helicase activity (Kusch et al. 2004). The role of TIP60 in DNA repair has been demonstrated in mutational studies, where TIP60 mutants lacking HAT activity elicit accumulated presence of DSBs following exposure to gamma radiation (Sapountzi et al. 2006). TIP60 regulates two key processes during DNA repair. First, its HAT activity promotes HR by facilitating recruitment of DNA repair factors to the sites of damage (Murr et al. 2006). Second, TIP60 mediates the exchange of phosphorylated H2A.v with unmodified H2A.v variants (Madigan 2002). Following DSB induction, HAT activity results in acetylation of lysine 5 on phosphorylated H2A.v. The ATPase subunit of TIP60 then catalyzes the replacement of acetylated phospho-H2A.v with unmodified H2A.v, enabling removal of phosphorylated H2A.v from damaged chromatin and thereby facilitating efficient DSB repair (Yeasmin and Torrente 2025).

Human hTIP60 is thought to function similarly by promoting the clearance of γH2AX from chromatin. hTIP60 accumulates at DSB sites containing γH2AX-modified nucleosomes, in a manner analogous to *Drosphila* TIP60 (Murr et al. 2007). Actin-related proteins (ARPs) are components of many chromatin remodeling complexes, and ARP4 enhances the interaction of the NuA4 complex with γH2AX. Likewise, the NuA4-related BAP55 subunit of the TIP60 complex may contribute to its recruitment to damaged DNA regions enriched in phospho-H2A.v (Downs et al. 2004). The p400 ATPase component of TIP60 facilitates the exchange of H2A.Z within nucleosomes at DSB sites, and its recruitment is dependent on γH2A.Z (Attikum et al. 2007). H2A.Z deposition at DSB sites is important for histone ubiquitination by RNF8 and acetylation of histone H4 by TIP60. H2A.Z has been reported to favor the NHEJ pathway through recruitment of Ku proteins, whereas its absence can lead to excessive DNA end resection and defective DSB repair (Xu et al. 2012).

With respect to tumorigenesis, TIP60 has been reported to function as a tumor suppressor by regulating the transcriptional activity of p53. However, breast carcinomas with reduced TIP60 expression often still exhibit p53 mutations, suggesting that TIP60 may exert tumor suppressor functions independent of p53 acetylation. Furthermore, p53 knockout mice with heterozygous TIP60 deletion exhibit enhanced mammary tumorigenesis. Reduced TIP60 levels have also been associated with impaired DNA repair in both tumor and normal tissues. In this context, TIP60 is implicated in regulating HR-mediated DDR, and its expression is inversely correlated with gene expression signatures associated with defective HR repair (Bassi et al. 2016; Zohourian et al. 2024). Together with the newly described role of BRD3 in cooperating with TIP60 to promote R-loop-mediated HR (Covre et al. 2015), these findings highlight TIP60 as a promising target whose modulation could sensitize tumors to DNA-damaging agents and PARP inhibitors.

#### SWR1

SWR1 is another multi-subunit chromatin remodeling complex with ATPase activity. It co-purifies with the histone variant H2A.Z, indicating a direct association with this histone variant. SWR1 is recruited to DSB sites through interaction with γH2A. The conserved histone variant H2A.Z (Htz1 in yeast) is deposited by SWR1 through exchange of the H2A-H2B dimer with the H2A.Z-H2B dimer in a unidirectional and stepwise manner (Luk et al. 2010). H2A.Z is widely distributed in yeast chromatin but is particularly enriched near transcription start sites of silent genes and at boundary regions separating heterochromatin from euchromatin. Research evidence implicates H2A.Z (Htz1) in DNA repair processes, and mutants lacking *SWR1* show increased sensitivity to DNA-damaging agents such as methyl methanesulfonate and hydroxyurea (Bao 2011; Morillo-Huesca et al. 2010). The presence of H2A.Z-containing nucleosomes promotes a more open chromatin configuration, facilitating nucleosome displacement while preventing the spreading of heterochromatin. In addition to its role in transcriptional regulation, SWR1 also participates in DNA repair by regulating γH2AX levels at DSB sites (Oberdoerffer and Miller 2023). It has been reported that in *pph3* mutants checkpoint adaptation occurs normally even though γH2AX levels remain elevated. This observation suggests that the requirement for γH2AX during checkpoint release may differ depending on whether DNA damage is repairable or persistent. Further, studies indicate that SWR1 incorporates H2A.Z at HO-induced MAT DSB sites in the absence of INO80, particularly when γH2AX levels are reduced. Recruitment of the RSC complex also facilitates the binding of yeast Ku80 (yKu80) and promotes NHEJ, suggesting that RSC and SWR1 may share partially overlapping roles in the NHEJ pathway (Attikum et al. 2007). More recently, the role of SWR1 in DDR has also been examined in *Arabidopsis thaliana*. Mutations in genes encoding components of the SWR1 complex render plants more sensitive to DNA-damaging agents (Rosa et al. 2013). Even in the absence of genotoxic stress, these mutants exhibit impaired DNA repair and accumulation of DNA damage. This defect is associated with reduced somatic HR. Interestingly, this phenotype contrasts with yeast SWR1 mutants, which display hyper-recombinogenic behavior, highlighting differences between higher and lower eukaryotes. Additional phenotypes observed in SWR1 mutants include fertility defects and impaired gametogenesis during meiosis. Collectively, these findings support a conserved role for SWR1 in somatic DDR and meiotic processes (Rosa et al. 2013). Although pharmacological inhibitors of SWR1 are not yet in clinical use, its regulation of H2A.Z deposition and γH2AX dynamics at DSB sites makes it a mechanistically relevant component of the cancer chromatin landscape with potential future therapeutic interest.

#### RAD54

RAD54 is a Swi2/Snf2-related, evolutionarily conserved dsDNA-stimulated ATPase and DNA translocase that functions as an important component of the HR pathway involved in DSB repair. RAD54 plays a key role in facilitating the recruitment of RAD51 to DNA damage sites and assists in regulating RAD51 activity during formation of the presynaptic filament (Solinger and Heyer 2001). In addition, RAD54 promotes branch migration of Holliday junctions and facilitates DNA synthesis by providing suitable substrates for DNA polymerases (Solinger et al. 2001). RAD51 can stimulate RAD54-mediated nucleosome remodeling in vitro, and its activity is essential for efficient strand invasion. These findings suggest that RAD54 function in the HR pathway is closely linked to chromatin remodeling. RAD54 activity is also dependent on its ATPase function (Wolner and Peterson 2005). Although the ATPase domain may not be required for presynaptic filament formation, it is necessary for the generation of stable DNA joint molecules. This observation suggests that chromatin is a key substrate for RAD54 primarily during the later stages of the HR repair pathway. The principal effect of RAD54-mediated chromatin remodeling is to increase DNA accessibility at the sites of repair. Supporting this notion, RAD54 has been shown to remodel a nucleosome positioned over the HO-induced cleavage site at the HMLa donor locus (Wolner and Peterson 2005). Thus, by enhancing DNA accessibility, RAD54 promotes the formation of DNA joint molecules and facilitates localized chromatin remodeling during HR repair without requiring large-scale nucleosome displacement. While RAD54 lacks approved clinical inhibitors, its essential role in RAD51-mediated HR suggests that pharmacological inhibition of RAD54 could induce HR deficiency, thereby sensitizing otherwise HR-proficient cancers to PARP inhibitors through synthetic lethality, warranting further investigation as a therapeutic target.

### Chromatin remodelers in cancer pathophysiology

The diverse pathophysiological implications of chromatin remodelers and their dysfunction in tumorigenic and metastatic pathways are only beginning to be fully understood. Advances in genome-wide sequencing technologies, including exome sequencing and genome-wide expression profiling in human cancers, have provided new opportunities to identify links between nucleosome remodeling complexes and cancer development (Weinstein 2002). Genetic mutations represent a major factor contributing to the transformation of normal cells into metastatic tumor cells. Many tumor-promoting gene expression alterations occur in the form of somatic mutations that enhance oncogene activity or suppress tumor suppressor gene expression.

Genetic mutations in ATP-dependent chromatin remodelers have recently been shown to influence cancer development and progression (Table 3). The SWI/SNF family of chromatin remodelers is among the most frequently mutated classes in human cancers (Kadoch et al. 2013). Genetic alterations in SWI/SNF subunits such as SMARCB1 (INI1) and ARID1A (BAF250A) have been associated with a wide range of malignancies, including cancers of the central nervous system, stomach, intestine, uterus, ovary, liver, and urinary tract. Another SWI/SNF subunit, BRG1 or SMARCA4, has also been reported to harbor mutations in cancers of the esophagus, intestine, lung, and urinary tract. In addition, PBAF-specific subunits such as BAF180 (PBRM1) and ARID2 are frequently mutated in kidney tumors.

**Table 3:** Summary of different chromatin remodelers and their subunits’ involvement in different types of cancer

Chromatin Remodelers	Subunit	Genomic alterations	Cancer type	Reference(s)
SWI/SNF	ARID1A (BAF250A), ARID1B/BAF250B, ARID2/BAF200	Loss-of-function mutations, inactivation mutations,	Endometroid and ovarian clear cell cancers, Colorectal cancer, Lung cancer	Mullen et al. 2021; Cajuso et al. 2014)
SWI/SNF	BRG1/BRM, SMARCA4/BRG1	Loss of BRG1/BRM, and inactivation	Lung cancers and adenocarcinoma, melanoma, myeloma, liver, bladder	Reisman et al. 2003; Concepcion et al. 2022)
SWI/SNF	BAF180	Overexpressed	Melanoma, Renal carcinomas	Pawłowski et al. 2013)
SWI/SNF	BAF53/BAF57	Knockdown results in the deregulation of important genes	Prostate and breast cancer	Balasubramaniam et al. 2013)
INO80	TRAAP/p400	knockdown results in increased tumor cell differentiation	Brain tumor	Wurdak et al. 2010)
INO80	RUVBL1/RUVBL2	Overexpressed	Hepatocellular carcinoma and colorectal cancer	Grigoletto et al. 2011)
CHD	CHD3/CHD4	frequently mutated	Cervix, stomach, endometrium Cancer, Prostate Cancer	Stanley et al. 2013)
CHD	MTA1	Overexpressed	Breast cancer	Li et al. 2010)
CHD	HDAC1/HDAC2	Overexpressed	T-cell lymphoma	Hagelkruys et al. 2011)
CHD	MBD2	frequently mutated	Colon, lung, prostate, and renal cancers	Magdinier and Wolffe 2001; Majid et al. 2009)
CHD	CHD5	Deletion	Cancer related to the CNS, hematopoietic system, and epithelium (thyroid, colon, cervix, and breast)	Bagchi et al. 2007)
CHD	CHD1/CHD2/CHD7	frequently mutated	Prostate cancer, Lung Cancer	Liu et al. 2012; Pleasance et al. 2010)
ISWI	SNF2H/SNF2L	under expressed	Breast cancer	Ye et al. 2009)
ISWI	BPTF	frequently duplicated	Neuroblastoma	Buganim et al. 2010)
NURF chromatin complexes	BAP18	Upregulated	TNBC	Zhang et al. 2022)
SWI/SNF	SMARCA4	Interacts with TGFB2-AS1	TNBC	Zhou et al. 2022)
SWI/SNF	BRG1	Upregulated	TNBC	Wu et al. 2016a)
SWI/SNF	ARID1A	Reduced expression	TNBC	Wang et al. 2024)

Recent studies have also highlighted the regulatory role of long noncoding RNAs (lncRNAs) in chromatin remodeling. For example, TGFB2-AS1 has been reported to negatively correlate with triple-negative breast cancer (TNBC) progression. TGFB2-AS1 interacts with SMARCA4, a SWI/SNF subunit, and interferes with its binding to target promoters, thereby limiting the expression of downstream genes (Zhou et al. 2022). The role of ARID1A (BAF250A), another component of the SWI/SNF complex, has also been investigated in TNBC pathology, where reduced ARID1A expression has been associated with specific breast cancer cell lines (Zhang et al. 2019). In addition, mutations in another chromatin modifier, lysine methyltransferase 2 C (KMT2C), have been reported in Thai clinical cases of TNBC (Niyomnaitham et al. 2019). The potential race- or population-specific associations between chromatin modifiers and cancer pathogenesis require further investigation.

Gene expression alterations in chromodomain helicase DNA-binding (CHD) ATPases, members of the SNF2 superfamily, have also been linked to several cancers. Subunits of other chromatin remodeling complexes, including ISWI and INO80, are similarly mutated or dysregulated in malignancies (Malone and Roberts 2024). For example, BAF53A, RUVBL1, and RUVBL2 components of the INO80 complex are overexpressed in bladder, cervical, myeloma, colon, and ovarian cancers. Likewise, nucleosome remodeling and deacetylase (NuRD) complex subunits such as MTA1, HDAC1-2, and RbAp48 have been reported to exhibit altered expression in cervical, pancreatic, leukemic, colon, and esophageal cancers. Expression levels of BAP18, a component of the NuRF remodeling complex, have also been reported to be altered in TNBC tissues. Mechanistically, BAP18 promotes cancer cell proliferation, migration, and invasion. Hyperactivation of BAP18 has been shown to enhance TNBC progression by activating transcription of the oncogene S100A9 (Zhang et al. 2022). Compared with other remodelers, expression alterations in the ISWI family appear to be less frequently associated with cancer pathophysiology, although overexpression has been reported in cancers of the head, cervix, and kidney.

### Impact of chromatin disorganization on cancer progression

Prior research has indicated that intrinsic heterogeneity within tumors strongly influences cancer progression and recurrence (Andrade et al. 2023). High-throughput genomic mapping of tumors has revealed the presence of multiple subclonal populations, with at least four distinct subclones identified in several cancers. This intra-tumor diversity can be further enhanced by chromatin rearrangements that occur during stochastic tumor growth or metastatic progression. Such alterations may influence gene expression through several mechanisms, including the formation of novel fusion transcription factors, the co-option of previously distal regulatory elements, or changes in epigenetic marks (Janiszewska 2020).

#### Tumor diversification due to chromatin disorganization

Apart from chromatin reorganization, altered nuclear positioning of chromosomes has been described in cancer cells both in vitro and in vivo (for example, chromosomes 18 and 19). Such changes in nuclear architecture may influence gene expression either through relocation of genes to different nuclear compartments or through increased mutation rates associated with these new nuclear environments (Cremer et al. 2003). Normally, chromatin associated with nuclear lamina is transcriptionally repressed. Consequently, genes relocated to this region often exhibit reduced expression in several model systems. Chromatin positioning also affects mutational rates. In general, chromatin located at the nuclear periphery displays a higher frequency of somatic mutations in cancer cells compared with chromatin positioned more internally within the nucleus. This observation suggests that peripheral chromatin may be more exposed to mutagens and may also undergo more error-prone DNA repair processes. For instance, approximately 40% of somatic mutations in several cancers occur within heterochromatic regions enriched in H3K9me3 (Sehgal and Chaturvedi 2023; Wu et al. 2019). In addition, many chromosomal translocations observed in cancers are highly specific. This specificity may arise because the corresponding loci are already spatially proximal within the nucleus, thereby increasing the probability of their fusion during DNA repair. Such events are particularly likely in heterochromatic regions, where the frequency of MMEJ is elevated (Schep et al. 2021). Furthermore, a subset of cancers has been shown to contain extrachromosomal DNA (ecDNA) particles. These elements can form extensive chromatin contacts due to the absence of normal higher-order chromatin compaction. As a result, ecDNA frequently drives high-level oncogene expression in multiple cancer types and is often associated with poor clinical prognosis (Wu et al. 2019).

#### Aneuploidy and resistance to therapy

The concept of chromosomal instability and the genetic heterogeneity of cancer cells bear similarities to the variability observed in yeasts, a group of asexual unicellular organisms. For instance, tetraploid yeasts evolve more rapidly than diploid yeasts under nutrient stress. Similarly, aneuploid cancer cells may experience fitness disadvantages due to imbalanced gene expression, yet they can gain proliferative advantages under stressful conditions such as hypoxia within the TME or exposure to therapeutic agents (Ben-David and Amon 2020). This phenomenon resembles Muller’s ratchet, which relates to the evolutionary accumulation of irreversibly deleterious mutations. Complex genomic rearrangements are frequently observed in aggressive tumors and may lead to gene disruption, formation of novel fusion proteins, or amplification of oncogenes. Only a small fraction of cells survive such extensive genomic rearrangements, and these surviving cells can contribute to tumor diversification. Consequently, clonal evolution and chemotherapy resistance may be driven by the persistence of “drug-tolerant” cancer cells that possess chromatin structures distinct from those of therapy-sensitive cells (Sehgal and Chaturvedi 2023; Rutledge et al. 2016).

#### Enhanced tumor invasiveness

Cell migration through tissue microenvironments is essential for processes such as development, immune surveillance, and tissue morphogenesis. During migration, nuclear positioning and morphology dynamically change, and these processes are partly regulated by chromatin compaction. Indeed, studies using drug-induced chromatin decondensation have shown reduced cell migration, although the precise contribution of chromatin compaction to this process remains under investigation. Increased chromatin compaction in response to migration cues has been described in fungi such as *Neurospora crassa* (Gerlitz et al. 2007). Similarly, during *Drosophila* embryogenesis, nuclear elongation is accompanied by chromatin condensation into a defined chromocenter, suggesting that chromatin compaction may regulate nuclear mechanics and stiffness during cell migration (Brandt et al. 2006). The heterochromatin protein 1 (HP1) family, including HP1α, HP1β, and HP1γ, plays a central role in mediating chromatin compaction (Kumar and Kono 2020). Loss of HP1α has been associated with increased metastatic potential in breast cancer cells due to disruption of peripheral heterochromatin tethering to the nuclear lamina, resulting in more deformable nuclei. This alteration may lead to increased chromosomal segregation errors and abnormal nuclear morphology (Sehgal and Chaturvedi 2023; Brandt et al. 2006). Chromatin stiffness mediated by HP1α is thought to arise from chromatin fiber bridging rather than from global increases in histone methylation levels (Strom et al. 2021; Beck and Hurt 2017). Thus, chromatin mechanics contribute significantly to maintaining nuclear stiffness and limiting metastatic potential. Interestingly, both decreased and excessive expression of HP1 isoforms can be detrimental for chromosomal stability (González-Barrios et al. 2014).

## Chromatin remodelers as potential biotargets for anticancer therapy

The cellular pathways responsible for repairing DNA damage can provide a growth advantage to cancer cells. These non-oncogenic dependencies are often difficult to define but represent attractive targets for cancer therapy. In recent years, pharmacological inhibitors targeting DDR pathways have been developed and evaluated to promote tumor cell death. Identifying differences in the utilization or activity of DDR pathways between tumor and healthy cells is a prerequisite for the development of effective repair inhibitors for targeting different carcinomas. The success of such therapeutic agents largely depends on selecting appropriate target proteins involved in repair pathways which are known to be critical regulators of the oncogenic processes. These proteins may contribute to cancer development due to genetic mutations that lead to overexpression or aberrant activity. Synthetic lethality can be exploited to selectively eliminate tumor cells by targeting complementary DNA repair pathways essential for cancer cell survival (Morandell et al. 2012). Two genes are considered synthetically lethal when inhibition of either gene alone permits cell viability, but simultaneous inhibition of both leads to cell death (Kaelin 2005). Therefore, identifying proteins with complementary roles in DNA repair pathways is essential for designing targeted therapies.

Epigenetic regulators have also emerged as promising therapeutic targets in several diseases, including cancer. For example, CHD4, a chromatin remodeler, has been identified as a potential therapeutic target in breast cancer, as its silencing has been shown to inhibit cancer cell proliferation. Cells harboring BRCA2 mutations together with reduced CHD4 expression exhibit shorter progression-free and overall survival (Guillemette et al. 2015). EZH2, a histone methyltransferase, represents another promising epigenetic target. Tumors with BRG1 loss-of-function mutations have been reported to respond to EZH2 inhibition. Conversely, EGFR- and BRG1-wild-type tumors may upregulate BRG1 in response to EZH2 inhibition and subsequently develop resistance to TopoII inhibitors. It has also been shown that ectopic expression of the BRG1 bromodomain can suppress γ-H2AX formation and DNA repair following irradiation, thereby increasing radiosensitivity in cancer cells (Kwon et al. 2015).

Bromodomains are present in several chromatin remodelers (Tallant et al. 2015), and specific inhibitors targeting these domains, including Baz2-ICR, GSK2801, and PFI-3, have been developed for bromodomains of BAZ2A/BAZ2B and BRG1 (Fedorov et al. 2015). Interestingly, PFI-3 inhibitors did not significantly affect the proliferation of TNBC cells. However, targeting the ATPase domain of BRG1 using the DNA-dependent ATPase A domain inhibitor (ADAADiN) resulted in a marked reduction in TNBC cell proliferation (Wu et al. 2016b). BRG1 is essential for breast cancer cell proliferation and is frequently overexpressed in breast tumors. Accordingly, BRG1 knockdown sensitizes TNBC cells to chemotherapeutic agents. Furthermore, CRISPR/Cas9-mediated deletion of BRG1 has been shown to impair cancer cell viability (Wu et al. 2016a). In combination with BRG1 knockdown, inhibitors targeting BRG1 bromodomains or ATPase activity have been shown to enhance the effectiveness of chemotherapeutic treatments. Inhibition of the ATPase domain of BRG1 also blocks the induction of ABC transporter genes. Since ABC transporters contribute significantly to chemoresistance by promoting drug efflux, targeting BRG1 enzymatic activity may enhance tumor chemosensitivity and improve therapeutic outcomes in breast cancer (Wu et al. 2016a).

The CHD1 gene, which encodes the chromodomain helicase DNA-binding protein 1, is frequently deleted in prostate cancer. Functional studies have shown that CHD1 plays a critical role in DSB repair by facilitating the recruitment of CtBP-interacting protein (CtIP) to chromatin and promoting DNA end resection. CHD1 contributes to chromatin opening at DSB sites, thereby enabling the recruitment of HR repair proteins. Importantly, CHD1 depletion selectively impairs HR-mediated DNA repair without significantly affecting NHEJ (Zhou et al. 2018; Kari et al. 2016). These findings suggest that CHD1 loss increases cellular sensitivity to PARP inhibitors and may therefore provide therapeutic opportunities. Similar to BRCA1/2 mutations in ovarian cancer, CHD1 deletion could potentially serve as a biomarker for stratifying prostate cancer patients and identifying individuals who may benefit from PARP inhibitor-based therapies (Kari et al. 2016). In endometrial and ovarian carcinomas, ARID1A is one of the most frequently mutated chromatin remodeler genes. As a tumor suppressor, ARID1A plays an important role in regulating gene expression required for maintaining cellular differentiation, homeostasis, and cell-cycle control, as well as in facilitating DDR. Loss of ARID1A function due to somatic mutations promotes tumor growth and dissemination. Consequently, considerable interest has emerged in developing synthetic lethality-based therapeutic strategies for ARID1A-mutated cancers, particularly following the success of PARP inhibitors in treating tumors with deficiencies in HR-mediated DNA repair (Mandal et al. 2022). Collectively, it appears that epigenetic modulators targeting the TME and ECM are potential therapeutic strategies (Zolota et al. 2021), highlighting the importance of chromatin remodelers in precision medicine approaches aimed at targeting multiple pathways involved in cancer development and progression.

### Merging immunotherapy and epigenetics to target cancer

In recent decades, targeting epigenetic mechanisms involved in tumorigenic and immune pathways has emerged as a promising strategy for cancer therapy. Several “epi-drugs” have already been approved by the FDA for clinical use. These agents target the epigenome with varying capacities to restore normal epigenetic regulation, thereby suppressing oncogenic signaling and modulating the TME. Immune system responses represent a key regulatory checkpoint considered in the therapeutic application of epi-drugs for different cancers. Various immunotherapeutic strategies have been developed for multiple cancer types, including breast cancer. These approaches include antibodies targeting tumor cells, adoptive T-cell therapies, cancer vaccines, and regulatory strategies aimed at modulating immune activation, such as those involving PD-1 signaling (Barzaman et al. 2020).

PD-1, a membrane receptor encoded by the *PDCD1* gene, acts as an inhibitory regulator of immune responses. During tumorigenesis, malignant cells may exploit immune regulatory mechanisms to evade immune surveillance, resulting in activation of resistance pathways and immune escape. Immune checkpoint inhibitors have therefore been developed to target PD-1 signaling and its ligands PD-L1 and PD-L2, as well as CTLA-4. Antibodies directed against PD-1 ligands expressed on tumor cells represent an effective approach to block inhibitory signaling pathways. PD-L1 blockade antibodies have been approved by the FDA for treatment of several cancers (Mc Neil and Lee 2025; Lin et al. 2024). CTLA-4, a homolog of CD-28, competes with CD-28 for binding to ligands expressed on antigen-presenting cells (APCs) (Gardner et al. 2014). The potential of CTLA-4 blockade to enhance antitumor immunity was first demonstrated by Chambers and colleagues (Chambers et al. 2001). Subsequently, monoclonal antibodies targeting CTLA-4 were developed and approved by the FDA for treatment of melanoma (Specenier 2016). These antibodies are currently being evaluated in clinical trials for additional cancer types. Overall, immune checkpoint inhibitors provide a powerful strategy for treating various carcinomas and have significantly transformed the landscape of cancer therapy. Under physiological conditions, immune checkpoints act primarily as inhibitory regulators that maintain immune tolerance by controlling the timing and magnitude of immune responses (Pardoll 2012).

Therapeutic strategies based on epigenetic markers may be particularly beneficial in cancers characterized by high mutation rates. For example, the potential of the nucleosome remodeling and deacetylase complex as a prognostic marker has been investigated in melanoma patients. Significant differences in mutation rates and immune microenvironments were observed depending on tumor grade, survival stage, and prognosis (Liu and Wang 2020). Differential activation of immune-associated pathways across tumor grades suggests their potential relevance for both experimental and clinical studies, including melanoma, leukemia, lymphoma, and breast cancer. In other malignancies such as hepatocellular carcinoma, the role of chromatin organization and chromatin remodelers is increasingly being explored. A recent study developed chromatin organization-related gene signatures (CORGs) to predict prognosis and responses to immune checkpoint inhibitors. The authors identified 339 CORGs, of which 186 were associated with hepatocellular carcinoma prognosis. A nomogram based on CORGs demonstrated improved differentiation and calibration compared with existing staging systems, highlighting their potential utility for clinical prediction models (Chen et al. 2022). Another study investigated the role of the transcription factor *BACH1* in glioblastoma and primary malignant brain tumors. BACH1 has been identified as a pro-tumorigenic transcription factor regulating multiple aspects of cancer progression, including metastasis, metabolic reprogramming, and self-renewal. Elevated BACH1 expression was associated with increased resistance to immune responses. Furthermore, BACH1 overexpression increased the expression of glioma-derived chemokines and modulated immune checkpoint signaling in vitro, suggesting its potential as a prognostic marker for glioblastoma (Yuan et al. 2022).

Epigenetic drugs targeting chromatin remodelers are increasingly investigated as agents capable of sensitizing tumors to immunotherapy by reversing mechanisms of immune escape. Combining epigenetic agents with immunotherapy therefore represents a promising therapeutic approach. Epigenetic drugs may enhance responses to immune checkpoint blockade through several mechanisms (Woods et al. 2015). This emerging field of immunopharmacology offers advantages over conventional cancer therapies, with potential applicability across multiple cancer types and the ability to modulate tumor-associated immune responses. It has been observed that HDAC inhibition in melanoma-bearing mice enhances PD-1–based immunotherapeutic responses (Woods et al. 2015). Combined inhibition of H3K27 methylation together with anti-CTLA-4 therapy significantly reduces tumor size compared with anti-CTLA-4 treatment alone, indicating that H3K27me3 inhibition may enhance checkpoint therapy efficacy. In addition, epigenetic drugs have been shown to increase T-cell infiltration following checkpoint blockade by removing epigenetic marks that suppress chemokine expression in lung and ovarian cancers (Peng et al. 2015; Zhang et al. 2016). In preclinical models, combination therapy using checkpoint inhibitors targeting CTLA-4 and PD-1 together with epigenetic agents such as 5-AzaC and entinostat effectively eliminated primary tumors in colorectal and metastatic breast cancer models (Kim et al. 2014). Furthermore, inhibitors targeting epigenetic enzymes such as histone methyltransferases (GSK126), bromodomains (JQ1), and histone demethylases (INCB059872) have been proposed as potential agents for enhancing immunotherapeutic responses (Zhang et al. 2025).

Despite their promise, some epigenetic drugs such as 5-Aza-CdR exhibit limitations, including short half-life, inactivation by cytidine deaminase in vivo, and hematopoietic toxicity. These factors restrict their use in combination therapies (Covre et al. 2015). To overcome these limitations, second-generation epigenetic agents, including the DNA hypomethylating agent SGI-110 (guadecitabine), are currently under evaluation. Other second-generation drugs such as vorinostat, panobinostat, and entinostat are also being investigated in combination with immunotherapeutic approaches. Table 4 highlights some of the ongoing clinical trials of epigenetic drugs as anti-cancer agents.

**Table 4:** Examples of some of epigenetic drugs that are in phase 1 clinical trials along with the disease conditions they are targeting

Epigenetic Drugs	Number of Clinical trials under progress	Description
5-Azacytidine	115	1. Fourth ventricular administration of immune checkpoint inhibitor (Nivolumab) and Methotrexate or 5-Azacytidine for recurrent medulloblastoma, ependymoma, and other CNS malignancies (NCT06466798) 2. Infusion of 5-Azacytidine into the 4th ventricle in children with recurrent brain tumor (NTC02940483) 3. 5-Azacitidine and Decitabine epigenetic therapy for myeloid malignancies (NCT04187703) 4. 5-Azacytidine and/or Nivolumab in resectable HPV-associated HNSCC (NCT05317000) 5. A phase II study of the combination of Azacitidine and Pembrolizumab for patients relapsed/refractory Hodgkin's lymphoma (NCT05355051)
Etinostat	03	1. Neoplasms glandular and epithelial, bronchial neoplasm (NTC02897778) 2. Testing a new anti-cancer drug combination, Entinostat, and ZEN003694, for advanced and refractory solid tumors and lymphomas (NCT05053971) 3. A phase I/II study of combination immunotherapy for advanced cancers including HPV-associated malignancies, small bowel, and colon cancers (NCT04708470)
Vorinostat	12	1. Children with relapsed solid tumor, lymphoma, or leukemia (NTC01422499) 2. Vorinostat in combination with chemotherapy in relapsed/refractory solid tumors and CNS malignancies (NCT04308330) 3. HDAC inhibitor Vorinostat in resistant BRAF V600 mutated advanced melanoma (NCT02836548) 4. Olaparib in combination with Vorinostat in patients with relapsed/refractory and/or metastatic breast cancer (NCT03742245) 5. A phase I/II study of Paclitaxel plus Carboplatin plus Vorinostat in recurrent ovarian cancer (NCT00772798)
Panobinostat	04	1. Patients with multiple myeloma (NTC04150289) 2. Study of combination of PIGEV before autologous stem cell transplant in patients with Hodgkin's lymphoma (NCT01884428) 3. Infusion of Panobinostat (MTX110) into the fourth ventricle in children and adults with recurrent medulloblastoma (NCT04315064) 4. Study to investigate outcome of individualized treatment in patients with metastatic colorectal cancer (NCT05725200)
Guadecitabine	02	1. Combination study of Guadecitabine/ASTX727 and Pembrolizumab (NCT02998567) 2. A study investigating SGI-110 in combination with Ipilimumab in unresectable or metastatic melanoma patients (NCT02608437)
Olaparib	149	1. Breast and ovary cancer (NTC04041128) 2. The effect of glucose level and diabetes mellitus on Ctrough of Olaparib (NCT05081765) 3. ATr inhibitor in combination with Olaparib/Durvalumab (MEDI4736) in gynaecological cancers with ARId1A loss or no loss (NCT04065269)

### Future perspectives

Although significant progress has been made in understanding the role of chromatin remodeling and DNA repair pathways in cancer biology, several challenges remain. Future studies should focus on identifying biomarkers that predict responses to inhibitors targeting chromatin remodelers and DNA repair proteins. A better understanding of the interactions between epigenetic regulation, immune signaling, and DDR cascades may enable the development of more precise therapeutic strategies. In this regard, lncRNAs have emerged as new players with the ability to interact with chromatin, proteins, and other RNAs to regulate essential cellular processes. In particular, their regulatory potential to alter the epigenetic machinery and recruit chromatin-modifying complexes to specific genomic loci is increasingly recognized as instrumental in determining transcriptional activity for driving normal development and tissue-specific gene expression, as well as in oncological pathophysiology. Therefore, they appear to be potent therapeutic targets for restoring the epigenetic landscape and abnormal gene expressions in cancers (Begolli et al. 2019). Further, integrating epigenetic drugs with immune checkpoint inhibitors and conventional therapies such as radiotherapy and chemotherapy may further improve treatment efficacy. Advances in genomic and epigenomic profiling are expected to facilitate patient stratification and support the development of personalized cancer therapies.

## Conclusion

DNA repair machinery can provide a growth advantage and contribute to drug resistance in cancer cells. Proteins involved in these repair processes therefore represent challenging yet attractive targets for the development of effective cancer therapies. Significant efforts have been made both to identify suitable targets and to develop inhibitors of DNA repair pathways. Understanding the mechanistic differences between DNA repair pathways in cancerous and normal cells may facilitate the development of more selective and less toxic therapeutic inhibitors. DNA repair pathways that normally protect the genome against DNA-damaging agents can be exploited as therapeutic targets in cancer treatment. Many proteins involved in these pathways are frequently upregulated or dysregulated in tumors, making them suitable candidates for targeted inhibition. Targeting chromatin remodelers involved in DNA repair represents an additional strategy for developing novel anticancer therapeutics. Exploiting synthetic lethal interactions may further enable selective targeting of tumor cells. Chromatin remodelers and DNA repair proteins whose functions are essential for tumor survival provide promising opportunities for selective cancer cell eradication. Research on inhibitors targeting chromatin remodelers and DNA repair pathways has expanded considerably in recent years. In addition, epigenetic drugs, including inhibitors of histone deacetylases and DNMTs, can enhance several components of the antitumor immune response, such as tumor-associated antigen expression, antigen processing, chemokine production, and immune checkpoint regulation. Together, these advances highlight the therapeutic potential of targeting chromatin remodeling and DNA repair mechanisms as part of integrated cancer treatment strategies.

## Data Availability

No datasets were generated or analysed during the current study.
